# Myeloid-driven immunosuppression in head and neck cancer: single-cell ATAC/RNA and spatial transcriptomic perspectives

**DOI:** 10.3389/fonc.2025.1693152

**Published:** 2025-12-18

**Authors:** Rui Luo, Jianzheng Yang, Zimeng Cao, Bing Li

**Affiliations:** 1 Department of Otorhinolaryngology, The First Affiliated Hospital of Chongqing Medical University, Chongqing, China; 2 Department of Otorhinolaryngology Head and Neck Surgery, Chongqing General Hospital, Chongqing University, Chongqing, China; 3 Department of Ultrasound, Chongqing Nan’an District People’s Hospital, Chongqing, China; 4 Department of Laboratory Medicine, Chonggang General Hospital, Chongqing, China

**Keywords:** head and neck squamous cell carcinoma, myeloid reprogramming, single-cell RNA/ATAC, spatial transcriptomics, checkpoint blockade, PD-1/PD-L1

## Abstract

Head and neck squamous cell carcinoma (HNSCC) remains a prevalent epithelial malignancy. Immune-checkpoint inhibitors have reshaped first-line therapy for recurrent/metastatic disease; yet durable benefit is confined to a subset, reflecting myeloid-centric mechanisms—SPP1^+^ TAM barriers, cDC1/IL-12 insufficiency, and CXCL8–CXCR1/2–driven neutrophil trafficking—distinct from, and complementary to, classical lymphoid exhaustion. In this review we summarize advances from single-cell RNA and ATAC profiling and spatial transcriptomics that resolve macrophage, dendritic-cell and neutrophil programs, and appraise translational opportunities spanning myeloid reprogramming, innate–adaptive combinations and spatial biomarkers. We also discuss enduring challenges—including HPV-status heterogeneity, limited assay standardization and a scarcity of predictive metrics—that temper implementation. By integrating myeloid-informed readouts (e.g., SPP1–TAM burden, cDC1 competency, serum IL-8) with PD-1–based regimens, EGFR-directed antibodies and myeloid checkpoints (CD47–SIRPα, PI3Kγ, CXCR1/2), emerging strategies aim to restore antigen presentation, improve lymphocyte trafficking and remodel tumor–stroma interfaces. Our synthesis provides an appraisal of the evolving landscape of myeloid-informed precision immuno-oncology in HNSCC and outlines pragmatic standards and avenues for clinical translation. We hope these insights will assist researchers and clinicians as they endeavor to implement more effective, individualized regimens.

## 1 Introduction

Head and neck squamous cell carcinoma (HNSCC) remains a common epithelial malignancy with heterogeneous etiologies and outcomes, including tobacco- and alcohol-associated disease and virally driven subsets (1–3), and immune checkpoint inhibitors have become part of first-line therapy for recurrent or metastatic disease on the basis of randomized evidence (4–7); nonetheless, durable benefit is restricted to a subset of patients, underscoring the need to resolve mechanisms of immune failure at tissue and single-cell resolution.

Across HNSCCs, myeloid lineages constitute a dominant—and markedly heterogeneous—fraction of the tumor microenvironment, varying by HPV status, anatomic site, and spatial niche, and are repeatedly linked to immunosuppression and adverse prognosis (8–10). Tumor-associated macrophages (TAMs) quantified by canonical markers correlate with inferior survival in aggregated cohorts, while mechanistic studies implicate macrophage programs in antigen presentation deficits and T-cell exclusion (11–13). Monocytic and granulocytic myeloid-derived suppressor cells (MDSCs) are enriched in HNSCC and dampen antitumor effector functions through arginase activity, nitric oxide and reactive oxygen intermediates, and checkpoint ligand expression; contemporary reviews in HNSCC synthesize these developmental routes and therapeutic entry points (14–16). Neutrophils also show context-dependent roles in HNSCC with functional states that range from cytotoxic to immunoregulatory, aligning with clinical observations that neutrophil phenotypes vary with stage and microenvironmental cues (17, 18). These data converge on a model in which myeloid circuits—rather than lymphoid scarcity alone—are primary architects of immune resistance.

Single-cell RNA sequencing (scRNA-seq) established the cellular framework for this model by resolving malignant programs, stromal elements, and discrete immune states in primary and metastatic HNSCC (19–21). Foundational datasets defined partial epithelial–mesenchymal transition trajectories in malignant epithelium, exhausted and progenitor-like T-cell states, and macrophage heterogeneity with ligand–receptor interactions that forecast immune dysfunction (22, 23). Subsequent scRNA-seq maps across HPV-positive and HPV-negative tumors show site- and virus-dependent shifts, with HPV+ disease enriched for B/Tfh/TLS and antigen-presenting/CXCL9+ macrophages, and HPV− lesions enriched for SPP1+/TREM2+ lipid-handling TAMs and CXCL8-high neutrophil/MDSC programs, informing biomarker recalibration (e.g., SPP1–TAM thresholds, PD-L1 CPS context) and therapy selection (EGFR-antibody or myeloid-modulating combinations) (24, 25). These atlases provide candidate axes for immunologic failure (e.g., SPP1^+^ macrophages, interferon-conditioned dendritic cells, CXCL chemokine circuits) that are testable in prospective cohorts.

Complementing transcriptomic views, single-cell chromatin accessibility profiling now captures cis-regulatory logic in HNSCC. In HNSCC myeloid cells, scATAC peak–gene links reveal accessible enhancers at SPP1/APOE–TREM2 and CIITA that track, respectively, with SPP1+ lipid-handling TAMs and antigen-presenting macrophages, while motif deviations for IRF/STAT, AP-1, C/EBPβ, PPAR/MAF and PU.1 align with polarization and CXCL8–CXCR1/2–neutrophil programs, with orthogonal evidence still required for causality (26–28). Broader single-nucleus ATAC-seq efforts across carcinomas validate the feasibility of mapping lineage-restricted enhancer usage and transcription-factor dependencies at single-cell resolution, enabling cross-cancer comparison of myeloid regulatory programs relevant to HNSCC (29–31).

Spatially resolved transcriptomic profiling has further demonstrated that the organization of myeloid, lymphoid, and malignant niches carries prognostic information and aligns with therapeutic responsiveness (32, 33). In HNSCC cohorts analyzed by single-cell-aware spatial methods, reproducible architectures—such as macrophage-dense tumor cores juxtaposed with immune-excluded epithelial fronts or myeloid-enriched stromal corridors—associate with survival and with inferred sensitivity to targeted and immune therapies (34, 35), indicating that spatial context is not incidental but determinative. Spatial analyses also connect early dissemination and nodal progression to immune-evasion ecosystems that feature coordinated myeloid–tumor signaling, reinforcing the translational value of mapping myeloid topology alongside malignant trajectories.

This review takes a myeloid-centric perspective on immunosuppression in HNSCC and synthesizes evidence from single-cell RNA and ATAC assays and spatial transcriptomic studies. The objectives are to delineate conserved and context-specific myeloid programs that constrain antitumor immunity; to map crosstalk between myeloid states and lymphoid dysfunction within spatial ecosystems; and to connect these features to emerging biomarkers and therapeutic strategies. Emphasis is placed on studies from Springer Nature and Elsevier journals to ensure methodological transparency and reproducibility. Examine tumor-associated myeloid programs in HNSCC at single-cell resolution, analyze spatially organized immune ecosystems and myeloid–lymphoid interactions, assess clinical translation through biomarker development and myeloid-directed interventions, and propose practical standards for integrative myeloid profiling in HNSCC.

## 2 Single-cell programs of tumor-associated myeloid cells in head and neck cancer

Single-cell profiling delineates recurrent myeloid states in head and neck squamous cell carcinoma (HNSCC) that are mechanistically linked to antigen presentation deficits, T-cell dysfunction, and malignant progression (36, 37). As shown in **Table 1**, early atlases established the cellular framework of the HNSCC microenvironment and revealed macrophage and dendritic-cell diversity across primary tumors and nodal metastases, with differences between HPV-positive and HPV-negative disease in compartment proportions and transcriptional programs (38, 39).

**Table 1:** Working definition and test results for tumor-associated myeloid status.

Entity/state	Canonical single-cell RNA markers	Single-cell ATAC/motif features (typical)	Common ligand–receptor axes (examples)	Functional readouts used in this review	Spatial tendency (descriptive)
SPP1^+^ tumor-associated macrophage (TAM)	SPP1, APOE, TREM2, MERTK, MARCO (variable)	Enrichment of AP-1, MAF/MAFB, PPAR motifs; accessible enhancers at lipid-handling genes	SPP1–CD44, TGFB1–TGFBR, VEGFA–KDR	Phagocytosis suppression, matrix remodeling indicators, HLA-II down-modulation	Tumor core and stromal corridors adjacent to invasive fronts
Antigen-presenting/CXCL9^+^ macrophage	CXCL9, CXCL10, HLA-DRA, CD74, IRF1	IRF/STAT motif accessibility; CIITA-linked enhancers	CXCL9/10–CXCR3, CD86–CTLA4/CD28	Antigen presentation score, interferon-stimulated gene score	Interface with T-cell aggregates or tertiary lymphoid structures
cDC1 (type-1 conventional DC)	XCR1, CLEC9A, BATF3, CADM1	IRF8/BATF3 motifs; cross-presentation module	XCL1/2–XCR1, CD40–CD40LG, IL12–IL12R	Cross-presentation and IL-12 production indices	Perivascular and lymphatic-adjacent niches; draining nodes
cDC2/mreg-DC (migratory LAMP3^+^ DC)	CD1C/FCER1A (cDC2); LAMP3, CCR7, CCL19/21, PD-L1/ICOSL (migratory)	RELB/NF-κB motifs; CCR7-associated enhancers	CCL19/21–CCR7, PD-L1–PD-1, ICOSL–ICOS	T-cell priming vs. tolerogenic polarization metrics	Stromal tracks leading to lymphatics; intratumoral T-cell clusters
Monocytic MDSC-like states	S100A8/A9, LILRBs, ARG1 (contextual), IL1B	C/EBP and STAT3 motif accessibility	CSF1–CSF1R, IL-1β–IL1R, TGFB–TGFBR	Arginase/ROS gene modules; T-cell suppression assays (*in vitro*)	Perivascular and immune-excluded regions
Neutrophil/TAN programs	CXCR2, FCGR3B, LTF, MMP9; immature–mature gradients	C/EBPβ, PU.1 motif accessibility	CXCL8–CXCR1/2, OSM–OSMR	Degranulation/NET signatures; chemotaxis indices	Border zones and necrotic areas; gradients from vessels
Assay notes (cross-modal)	RNA: state identity; ATAC: cis-regulatory logic; spatial: niche mapping	—	—	Composite scores harmonize across modalities	“Spatial tendency” is descriptive; not a prognostic assignment

Macrophages constitute the dominant myeloid compartment and separate along axes of SPP1^+^ lipid-handling, matrix-remodeling TAMs versus interferon-conditioned, antigen-presenting CXCL9/10^+^ states (40, 41). In HNSCC, SPP1^+^ TAMs show functional coupling to tumor and stromal programs and are linked to invasive phenotypes, while antigen-presenting macrophages co-localize with T-cell aggregates and tertiary lymphoid features (42, 43). Single-cell studies and integrative analyses in HNSCC demonstrate that SPP1+ TAMs engage CD44 and NF-κB–linked pathways, under PI3Kγ–AKT–CREB/STAT3 control, and form reciprocal interactions with SFRP2+ cancer-associated fibroblasts, supporting a pro-invasive ecosystem (44, 45).

Dendritic-cell programs bifurcate along an immature→mature continuum: pre-cDC1 (IRF8^high^/BATF3^+^) mature into XCR1^+^CLEC9A^+^ cDC1 that cross-present and deliver IL-12, whereas cDC2 transition to LAMP3^+^CCR7^+^ migratory ‘mreg-DC’ that upregulate PD-L1/ICOSL and acquire immunoregulatory secretomes (46, 47). In HNSCC models that preserve tumor-draining lymphatics, cDC1 accumulation and type-I-interferon signaling are necessary for response to checkpoint blockade; disruption of lymphatic egress abrogates these features and diminishes efficacy (48, 49). Single-cell phenotyping also links a PD-L1^high^ ICOSL^low^ “secretory” DC state to attenuated T-helper polarization, whereas ICOSL-dominant DC associate with improved immune activation, including in HNSCC cohorts.

Myeloid-derived suppressor phenotypes emerge along monocytic and granulocytic lineages, with monocytes (LYZ^+^, S100A8/A9^+^) differentiating toward SPP1^+^ TAMs or antigen-presenting-like macrophages, and neutrophils progressing from immature CXCR2^+^/LTF^+^ states to antigen-presenting-like, MHC-II^+^/CD74^+^ subsets. Pan-cancer single-cell integrations identify conserved TREM2^+^/FOLR2^+^ myeloid states and clarify that suppressive programs can be misclassified when analyzed in bulk; within HNSCC datasets these states co-occur with hypoxia and cytokine-rich niches (50, 51). In parallel, neutrophil programs span immature, inflammatory, and antigen-presenting-like trajectories; single-cell compendia across tumors, including head and neck cancers, resolve transcriptional continua that explain prior context-dependent associations of tumor-associated neutrophils with either immune control or suppression.

Transcriptional states are underpinned by cis-regulatory logic that is now measurable in HNSCC using single-cell and bulk ATAC-seq. Under EGFR blockade, enhancer remodeling occurs rapidly in epithelial and myeloid compartments, with IRF/STAT and AP-1 network shifts that presage adaptive resistance and track with interferon and antigen-presentation programs (52, 53). These observations support integrating single-cell ATAC with RNA to resolve lineage dependencies and to nominate tractable regulators of myeloid polarization in HNSCC.

Single-cell RNA and ATAC assays in HNSCC converge on a structured view of tumor-associated myeloid biology: SPP1^+^ macrophages and suppressive MDC/MDSC modules organize immune-excluded niches; cDC1 insufficiency and migratory LAMP3^+^ DC reprogramming constrain priming; and neutrophil states reinforce chemokine and matrix circuits. These axes provide testable links to spatial organization.

## 3 Spatial immune ecosystems and myeloid–lymphoid crosstalk

In HNSCC, spatial profiling shows TLS-rich lymphoid hubs and CD8–cDC1 corridors associate with survival and checkpoint response, whereas continuous SPP1+ TAM–tumor borders and POSTN+ CAF corridors mark immune exclusion and worse outcomes, indicating that topology adds prognostic and pharmacodynamic information beyond bulk composition. Single-cell–aware multiplex tissue imaging in human papillomavirus–negative tumors links greater compartmentalization of tumor–immune territories and mesenchymal neighborhood organization with improved progression-free survival, indicating that spatial topology is an informative dimension of immune competence in this disease (54, 55). Complementary spatial proteogenomic analyses in clinical specimens further show that heterogeneous tumor hubs are flanked by immune niches with distinct ligand–receptor activity, supporting the use of multi-omic spatial readouts to nominate therapeutic targets within location-defined ecosystems (56, 57).

Humoral–cellular immune hubs are prominent features of favorable ecosystems. In HPV-positive tumors, B-cell–rich aggregates with germinal-center architecture and T-follicular-helper activity form tertiary lymphoid structures that correlate with superior outcomes, where local antigen presentation, Tfh-guided affinity maturation, and HEV-mediated trafficking sustain CD8 and memory responses (58, 59). Distance-based spatial metrics generalize these observations: in pre-operative ipilimumab+nivolumab cohorts, nearest-neighbor relationships—rather than density—discriminate response; a pragmatic schema uses median CD8→cDC1 and CD8→HEV distances and macrophage–tumor interface length as spatial biomarkers of checkpoint benefit, derivable from multiplex IHC/CODEX or MIBI (60–62).

Myeloid positioning at tumor–stroma interfaces provides a mechanistic substrate for immune exclusion and checkpoint signaling. Spatial interaction mapping that resolves *in situ* PD-1/PD-L1 ligation identifies macrophage-dense layers at tumor margins in non-responders, consistent with a barrier that restricts effective lymphocyte access; in contrast, responders show B- and T-cell aggregates at tumor edges with fewer continuous macrophage–tumor interfaces (63–65). These findings align with single-cell and spatial views in oral cavity tumors in which SPP1^+^ tumor-associated macrophages co-localize with POSTN^+^ fibroblasts and malignant programs to form a desmoplastic, immunoregulatory network capable of shaping T-cell trafficking and function (66, 67), offering a tractable axis for ecosystem remodeling. For clinical sampling, prioritize cores that include the tumor–stroma interface and perivascular regions (and nodal metastases when present), because these sites maximize measurable myeloid–lymphoid adjacency and reduce false-negative proximity scores.

Antigen-presenting cell geography further constrains priming and effector function. Across carcinomas, tumor-retained CCR7^+^ migratory dendritic cells downregulate antigen-presentation programs with dwell time, implying that retention within the tumor parenchyma diminishes cross-priming potential (68, 69). Lymphatic-localized crosstalk between mature, immunoregulatory LAMP3^+^ dendritic cells and regulatory T cells has been shown to limit CD8^+^ T-cell immunity, highlighting a spatially delimited checkpoint that likely intersects with the architectures observed in head and neck cancer (70–72). Embedding these dendritic-cell circuits within HNSCC spatial atlases will clarify whether restoring cDC1 access to T cells within peritumoral or intratumoral niches can convert excluded to inflamed phenotypes.

These data indicate that spatial ecosystems in head and neck cancer are defined by adjacency rules—macrophage–tumor interfaces at borders, TLS-like lymphoid hubs, and dendritic-cell corridors linking tissue and lymphatics—that govern myeloid–lymphoid communication, checkpoint engagement, and therapeutic responsiveness. Mapping and perturbing these arrangements provide a pathway to convert suppressive architectures into productive antigen-presentation and T-cell–effector niches, and justify spatially informed biomarkers for clinical decision-making in this disease.

## 4 Clinical translation: biomarkers and myeloid-directed therapeutics

Clinically deployed biomarkers in head and neck squamous cell carcinoma currently prioritize PD-L1 combined positive score to select regimens containing pembrolizumab, yet response heterogeneity underlines the need for composite models that quantify myeloid-driven suppression in parallel with adaptive immune competence (73, 74). Randomized evidence demonstrated overall-survival benefit with pembrolizumab-based therapy in recurrent/metastatic disease and established PD-L1 thresholds for treatment assignment, but predictive performance remains incomplete, supporting development of adjunct biomarkers that index myeloid state and spatial organization (75–77).

**Figure 1:**
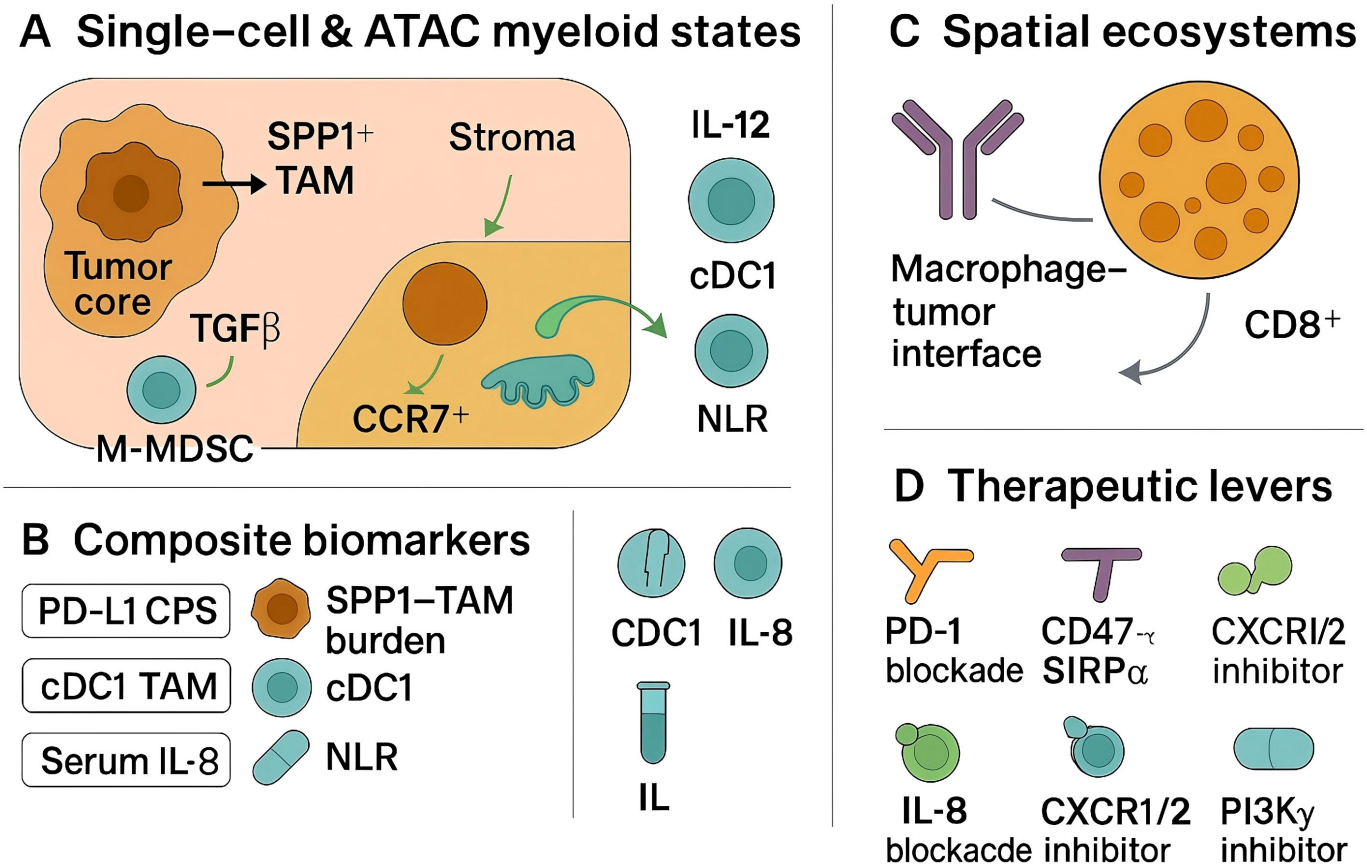
Myeloid-driven immunosuppression in HNSCC: single-cell states, spatial interfaces, and therapeutic levers.

Myeloid-informed tissue biomarkers are increasingly tractable. Single-cell–grounded signatures and immunohistochemical surrogates for SPP1^+^ tumor-associated macrophages correlate with immunosuppressive programs, matrix remodeling, and inferior outcomes in head and neck and oral cavity cohorts, nominating an “SPP1–TAM burden” as a candidate readout to complement PD-L1 and tumor mutation burden in clinical decision-making (78, 79). Quantification can be implemented in routine tissue: compute XCR1^+^CLEC9A^+^ cDC1 density (cells/mm^2^ by multiplex IHC) and an IL-12 module score from RNA panels (IL12A/IL12B with CXCL9/CXCL10 co-expression), alongside an SPP1–TAM burden (SPP1/CD68/MERTK IHC or RNA) (80, 81). As shown in **Figure 1**, circulating inflammatory signals that reflect myeloid trafficking also carry prognostic and pharmacodynamic information; baseline serum interleukin-8 predicts inferior benefit across anti–PD-1/PD-L1 and anti–CTLA-4 regimens (and PD-1–cetuximab combinations), consistent with its role in neutrophil/MDSC recruitment, supporting inclusion in pre-treatment risk stratification and on-therapy monitoring (82, 83). Readily available systemic indices, such as the neutrophil-to-lymphocyte ratio, have shown association with pembrolizumab efficacy in real-world head and neck cohorts and may provide cost-efficient triage when high-parameter profiling is not feasible (84, 85). Spatial biomarkers derived from tissue imaging and spatial transcriptomics further refine risk: tertiary lymphoid structures and B-cell–rich aggregates are reproducibly associated with favorable outcomes in head and neck cancer and can be co-analyzed with myeloid topology to contextualize checkpoint signaling and antigen presentation (86, 87). Preservation or restoration of cDC1-linked migratory circuits between tumor and draining lymphatics appears necessary for effective checkpoint responses, providing a mechanistic axis for spatially informed stratification.

Therapeutic strategies that directly target myeloid checkpoints or reprogram suppressive myeloid circuits are entering head and neck–relevant clinical testing. CD47–SIRPα blockade enhances macrophage-mediated phagocytosis; the high-affinity CD47 inhibitor evorpacept has demonstrated manageable safety and signs of activity across solid tumors including a head and neck expansion, and its biology supports combination with PD-1 inhibitors and with Fc-active EGFR antibodies used in this disease (88, 89). Dual targeting of the adaptive and innate compartments is further supported by pembrolizumab plus cetuximab: cetuximab engages FcγRIIIa on NK cells (ADCC) and FcγR on macrophages (ADCP), while PD-1 blockade reinvigorates effectors; however, macrophage ‘walls’ at tumor margins and FcγR polymorphisms may modulate benefit, motivating assays of myeloid exclusion and Fc competence (90, 91). Neutralization of IL-8 is a rational adjunct to checkpoint blockade given its association with primary resistance and myeloid chemotaxis; translational and preclinical data indicate that IL-8 axis inhibition can diminish suppressive myeloid infiltration and restore antitumor immunity (92, 93). TAM reprogramming via PI3Kγ inhibition has strong mechanistic support—PI3Kγ functions as a switch enforcing immunosuppressive transcriptional programs in myeloid cells—and represents a complementary route to enhance checkpoint efficacy, though definitive head and neck–specific clinical benefit remains to be established. Additional upstream interventions that curb myeloid recruitment, such as blockade of CXCR2-dependent neutrophil trafficking, are justified by preclinical evidence and early translational studies and warrant head and neck–focused evaluation with prespecified myeloid pharmacodynamic endpoints (94, 95). Emerging evidence in human papillomavirus–negative head and neck models also supports combined disruption of inflammatory cytokine and monocyte-chemokine pathways—for example, IL-6 plus CCR2 blockade—to limit suppressive myeloid niches and potentiate cytotoxic effector function (96, 97).

Implementation should prioritize analytically validated assays, predefined thresholds, and prospective integration into trial designs that randomize myeloid-directed combinations against current standard immunotherapy backbones. A pragmatic biomarker panel would pair PD-L1 CPS with a myeloid score (e.g., SPP1–TAM burden), serum IL-8, and a spatial readout capturing myeloid–lymphoid adjacency or tertiary lymphoid structure status, with adaptive sampling to evaluate on-treatment myeloid plasticity. Such schema allow attribution of benefit to myeloid modulation, enable early stopping for futility when myeloid targets are not engaged, and create a pathway to deploy myeloid-directed therapeutics in a biologically selected subset of patients with head and neck cancer.

## 5 Outlook and standards for integrative myeloid profiling in head and neck cancer

Integrative myeloid profiling in head and neck squamous cell carcinoma should advance from descriptive atlases to standardized, decision-oriented pipelines that co-register single-cell RNA, single-cell ATAC, and spatial readouts with harmonized metadata and predefined endpoints. Foundational single-cell and chromatin studies justify a state dictionary anchored on SPP1^+^ tumor-associated macrophages, antigen-presenting/CXCL9^+^ macrophages, cDC1, LAMP3^+^ migratory dendritic cells, monocytic/granulocytic MDSC, and context-dependent neutrophil programs, with motif-level regulators (IRF/STAT/AP-1) reported alongside ligand–receptor inferences (26–31, 40–47, 50, 51).

Spatial analyses indicate that adjacency metrics—macrophage–tumor interface length, tertiary lymphoid structure burden, and nearest-neighbor distances between myeloid and lymphoid cells—capture clinically relevant architecture beyond cell fractions and should be quantified with assay-specific quality controls (32–35, 58–62, 86, 87). For clinical translation, composite models should pair PD-L1 combined positive score with a myeloid score (e.g., SPP1-TAM burden), serum IL-8 and neutrophil-to-lymphocyte indices, and a spatial topology score, all benchmarked to response and survival under contemporary immunotherapy backbones (73–85).

Prospective trials should embed these assays with prespecified analytical thresholds, transparent batch correction, cross-platform validations, and on-treatment pharmacodynamic criteria that attribute benefit to myeloid modulation (depletion/reprogramming of suppressive states, restoration of cDC1 circuits, remodeling of macrophage–tumor borders) (46–49, 78–81).

We anticipate that HPV-negative tumors with high SPP1-TAM burden and continuous macrophage–tumor interfaces will be candidates for TAM-directed or IL-8–axis combinations layered onto PD-1 regimens, whereas HPV-positive, TLS-rich ecosystems may preferentially benefit from strategies that preserve cDC1 trafficking and enhance dendritic-cell priming (24, 25, 58, 59, 86, 87). Establishing these reporting and performance standards will enable reproducible risk stratification, facilitate cross-study synthesis, and accelerate deployment of myeloid-informed precision therapy in head and neck cancer.
