# Correction: Myeloid-driven immunosuppression in head and neck cancer: single-cell ATAC/RNA and spatial transcriptomic perspectives

**DOI:** 10.3389/fonc.2026.1788227

**Published:** 2026-03-06

**Authors:** Rui Luo, Jianzheng Yang, Zimeng Cao, Bing Li

**Affiliations:** 1 Department of Otorhinolaryngology, The First Affiliated Hospital of Chongqing Medical University, Chongqing, China; 2 Department of Otorhinolaryngology Head and Neck Surgery, Chongqing General Hospital, Chongqing University, Chongqing, China; 3 Department of Ultrasound, Chongqing Nan’an District People’s Hospital, Chongqing, China; 4 Department of Laboratory Medicine, Chonggang General Hospital, Chongqing, China

**Keywords:** head and neck squamous cell carcinoma, myeloid reprogramming, single-cell RNA/ATAC, spatial transcriptomics, checkpoint blockade, PD-1/PD-L1

Affiliation “Department of Ultrasound, Chongqing Nan’an District People’s Hospital, Chongqing, China” was omitted for author Jianzheng Yang. This affiliation has now been added for author Jianzheng Yang.

Author Jianzheng Yang was erroneously assigned to affiliation “Department of Otorhinolaryngology, The First Affiliated Hospital of Chongqing Medical University, Chongqing, China”. This affiliation has now been removed for author Jianzheng Yang.

Author Jianzheng Yang was erroneously assigned to affiliation “Department of Otorhinolaryngology Head and Neck Surgery, Chongqing General Hospital, Chongqing University, Chongqing, China”. This affiliation has now been removed for author Jianzheng Yang.

Author Zimeng Cao was erroneously assigned to affiliation “Department of Ultrasound, Chongqing Nan’an District People’s Hospital, Chongqing, China”. This affiliation has now been removed for author “Zimeng Cao”.

Author Bing Li was erroneously assigned to affiliation “Department of Laboratory Medicine, Chonggang General Hospital, Chongqing, China”. This affiliation has now been removed for author Bing Li.

The original version of this article has been updated.

